# Internet-based interventions for eating disorders in adults: a systematic review

**DOI:** 10.1186/1471-244X-13-207

**Published:** 2013-08-06

**Authors:** Ruth Dölemeyer, Annemarie Tietjen, Anette Kersting, Birgit Wagner

**Affiliations:** 1 Department of Psychosomatic Medicine and Psychotherapy, University of Leipzig, Semmelweisstr. 10, Leipzig 04103, Germany; 2 Leipzig University Medical Center, IFB AdiposityDiseases, Leipzig, Germany

**Keywords:** Eating disorders, Internet-based intervention, Systematic review, Binge eating disorder, Bulimia nervosa, EDNOS

## Abstract

**Background:**

This systematic review evaluates the efficacy of internet-based interventions for the treatment of different eating disorders in adults.

**Method:**

A search for peer reviewed journal articles detailing Randomised Control Trials (RCT) and Controlled Trials (CT) addressing participants with eating disorders aged at least 16 was completed in the electronic databases Web of Science, PsycInfo and PubMed. The quality of the included articles was assessed, results were reviewed and effect sizes and corresponding confidence intervals were calculated.

**Results:**

Eight studies, including a total of N = 609 participants, fulfilled the selection criteria and were included. The majority of treatments applied in these studies were based on CBT principles. Six studies described guided self-help interventions that showed significant symptom reduction in terms of primary and secondary outcomes regarding eating behaviour and abstinence rates. These studies produced significant medium to high effect sizes both within and between the groups after utilisation of guided self-help programs or a self-help book backed up with supportive e-mails. The two remaining studies utilised a specific writing task or e-mail therapy that did not follow a structured treatment program. Here, no significant effects could be found. Treatment dropout rates ranged from 9% to 47.2%. Furthermore, reductions in other symptoms, for example depression and anxiety, and an increase in quality of life were found by four studies.

**Conclusions:**

Overall, the results support the value of internet-based interventions that use guided self-help to tackle eating disorders, but further research is needed due to the heterogeneity of the studies.

## Background

Eating disorders are associated with both high social and personal costs for the person concerned. Most people with eating disorders do not access effective treatment [1] and show a preference for low-threshold interventions rather than conventional health care provided for mental health problems [2,3]. For this reason, an increasing number of internet-based interventions addressing eating disorders have been developed to facilitate access to effective treatments for these individuals. Many of these internet-based programs have been developed with the aim of preventing eating disorders [3-11], but more recently there has also been an increase in interest in internet-based interventions targeting people who already suffer from a diagnosed eating disorder [12-19]. While internet-based interventions addressing bulimia nervosa [14-19], binge eating [12,13,16], EDNOS (eating disorder not otherwise specified) [17,19] and body dissatisfaction [20,21] have been conducted, there is still a relatively low number of investigations into the application of internet-based interventions for anorexia nervosa. It can be assumed that this is due to the fact that the weight loss accompanying anorexia nervosa can be life threatening, making it the eating disorder with the highest mortality rate [22].

In general, internet-based interventions have several advantages, for example the lack of geographic boundaries, enabling widespread dissemination of treatment [23]. Furthermore, internet-based interventions are cost-effective [24] and provide greater user control, flexibility, open access and anonymity [25]. They are therefore especially relevant for patients who might not otherwise access treatment for reasons such as fear of social stigma or lack of easy access to a treatment centre.

While a number of reviews and meta-analyses of internet-based interventions have been published, e.g. for depression [26,27], depression and anxiety [28-31], obesity [32] and the prevention of eating disorders [3], there is, to our knowledge, no review examining the efficacy of internet-based interventions for the treatment of existing eating disorders. Thus, with this systematic review we aim to give an overview of the different forms of internet-based interventions that have been applied for people suffering from an eating disorder.

The following key questions are addressed by this systematic review:

1. What is the evidence for the value of internet-based interventions for the treatment of eating disorders? To answer this question, effect sizes and corresponding confidence intervals were calculated for within and between group analyses. Additionally, rates of abstinence and dropout were taken into account.

2. What factors are associated with these treatment effects (e.g. duration of treatment, degree of therapist involvement)?

To answer these questions only analyses of quantitative data were taken into account; qualitative data were not considered at this time.

## Methods

Before starting with literature search for the systematic review, inclusion criteria and methods of analysis were specified. These criteria have not been documented in an official review protocol.

### Study eligibility

Studies were selected and included in the present review according to five criteria: (1) publication in a peer reviewed journal, (2) presence of a controlled design, (3) inclusion of the internet as at least one mode of delivery for treatment or self-help, (4) inclusion of participants aged at least 16 years and suffering from an eating disorder and (5) presence of changed eating behaviour as a primary outcome. Studies were excluded if they (1) addressed prevention of eating disorders, (2) addressed weight loss programs, or (3) did not include subjects suffering from a diagnosed eating disorder.

### Study selection

For the selection of studies, Web of Science, PsycInfo and PubMed were electronically searched for articles published or e-published before November 2012 by combining the terms “random*” OR “controlled” with the terms “eating disorder”, “anorexia”, “bulimia”, “binge eating” OR “EDNOS”, the terms “online”, “internet”, “computer*”, “email” OR “web” and the terms “intervention”, “therapy” “self-help”, “treatment” OR “program” in titles or abstracts. No limitation was made regarding the language of articles. The titles and abstracts of the 460 articles identified by the initial search were screened to determine their relevance to the review. Articles that did not meet inclusion criteria were excluded at this stage, whereas the full text of potentially relevant studies was examined. Furthermore, the reference lists from retrieved articles were checked for additional relevant literature. The selection of articles was independently performed by the primary and secondary author of this review.

### Data extraction

Data extraction was conducted independently by two authors, consulting a third reviewer in the case of discrepancy in the documentation of study features. Variables extracted included the authors of the study, title and publication year, information regarding number, diagnosis, gender and age of participants, characteristics of intervention and control groups (e.g. frequency of contact, kind of control group), duration of treatments, time points of assessment, and measures used for outcome assessment. The rates of study dropouts (regarding percent of missing post-treatment assessments over all groups) and treatment dropouts (regarding percent of participants not finishing treatment in the intervention group) were also noted, as were rates of abstinence. Furthermore, information necessary for evaluating methodological quality was extracted.

### Assessment of methodological quality

Methodological quality was assessed using an 11-item list oriented on a scale developed by van den Berg et al. [33]. Studies were rated independently by the first author and checked by the second author. Disagreements were discussed until consensus was reached. Each item was rated as *yes*, *no*, or *unknown*. A total methodological quality score (ranging from 0–11) was calculated by summing up all *yes* items. Studies were rated as having good methodological quality if they met at least two-thirds of the criteria (eight or more items).

### Assessing the effects of internet-based interventions for eating disorders

To answer the key questions of this review, key eating disorder-related symptoms (e.g. bingeing and purging) and rates of abstinence were considered as primary outcomes. As secondary outcomes, results of key questionnaires were considered. Included were questionnaires that are commonly used for the assessment of symptoms related to eating disorders, resulting in examination of (1) EDE interview and the Eating Disorder Examination Questionnaire (EDE-Q) [34], a semi-structured interview and its self-report version, measuring the core psychopathology of eating disorders; and (2) the Eating Disorder Inventory (EDI) and Eating Disorder Inventory 2 (EDI-2) [35-38], which were developed to assess psychological characteristics of patients with eating disorders. In studies that did not include one of these questionnaires, (3) the Bulimia Investigatory Test Edinburgh (BITE) [39], a self-rating measure assessing symptoms of BN, was considered.

If a study measured outcomes across several time points, the first time point after completion of the intervention was selected for comparison of studies. For studies that included more than one control group, the one with the least contact was selected for comparison. Since statistical significance of t-tests depends upon sample size, level of significance, tests used and other variables of study design, effect sizes (ES, Hedges’ g) were calculated by the authors, according to Hedges [40]. For effect size calculation, intention to treat (ITT) data was used. Where ITT data were not available, ITT effects were estimated, assuming a zero effect for study dropouts. If no data for calculating or estimating effect sizes were available, results of ANOVAs or effect sizes reported in the studies were used. An ES of less than 0.5 was interpreted as small, 0.5 to 0.8 as medium and greater than 0.8 as large [41]. Additionally, 95% confidence intervals (CIs) were calculated according to Hedges and Olkin [40]. Furthermore, where follow-up data were available, stability of the effects was reported and additional outcomes (e.g. depression, anxiety or quality of life) were considered. Here, the (1) Beck Depression Inventory (BDI) [42], or BDI-II [43], (2) the Hospital Anxiety and Depression Scale (HADS) [44,45] or (3) the Montgomery Åsberg Depression Scale Self-assessment (MADRS) [46] were used to assess symptoms of depression and anxiety. To assess quality of life, (1) the short form of the Impact of Weight on Quality of Life (IWQOL-Lite) [47], (2) the Satisfaction with Life Scale (SWLS) [48] and (3) the WHO Quality of Life Questionnaire (WHOQOL-Bref) [49] were included in the review.

Because the studies differ in terms of the eating disorders addressed, characteristics of participants and internet-based programs applied, no meta-analysis was performed but results will be presented for each study and conclusion will be drawn.

## Results

### Study selection

**Figure 1:**
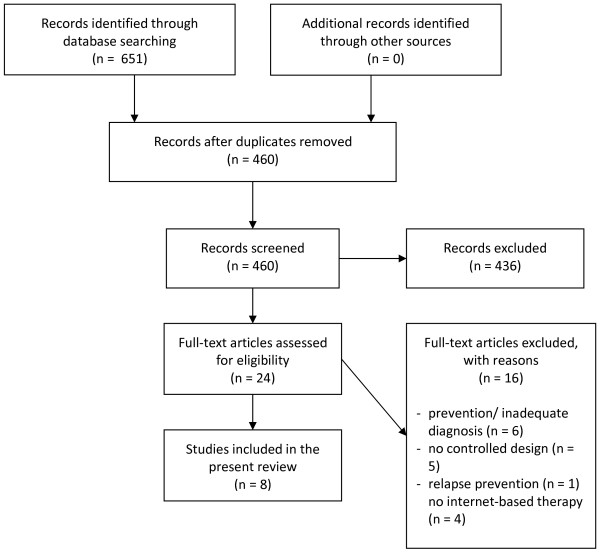
Identifying studies for inclusion in systematic review.

The search and selection process for articles is illustrated in Figure 1. A total of 651 articles were identified by the initial search. After removing duplicate articles (n = 191) and irrelevant studies (n = 436), 24 articles were retained for further consideration. Of these, 16 articles were excluded as they addressed prevention of eating disorders or presented data from participants who did not fulfil a diagnosis of an eating disorder (n = 6), they did not use a controlled design (n = 5), they were addressing relapse prevention (n = 1) or they did not use internet-based therapy as the mode of delivery (n = 4). Screening the reference lists from retrieved articles did not lead to the inclusion of any additional relevant literature → Figure 1.

### Methodological quality

In Table 1 the results of the methodological quality assessment are described. Of the eight studies included, five [12,16-19] were rated as having good methodological quality, whereas two studies [13,15] just missed this rating by meeting seven rather than eight out of the 11 criteria. One study [14] failed to report the eligible criteria and timing of outcome measurements between groups were not comparable.

**Table 1:** Assessment of methodological quality

**Items**	**Study**	**Ljotsson et al. **[16]	**Robinson & Serfaty [**[17]	**Johnston et al. **[15]	**Carrard et al. **[12]	**Sanchez-Ortiz et al. **[19]	**Fernandez-Aranda et al. **[14]	**Carrard et al. **[13]	**Ruwaard et al. **[18]
**Methodological quality**									
Were the eligible criteria specified?		yes	yes	yes	yes	yes	no	yes	yes
Was the method of randomization described?		yes	yes	no	yes	yes	yes	yes	yes
Were the groups similar at baseline regarding important prognostic indicators?		yes	yes	unknown	yes	yes	yes	yes	yes
Were the index and the control interventions explicitly described?		yes	yes	yes	yes	yes	yes	yes	yes
Was the outcome assessor blinded to the interventions?		Unknown	yes	Unknown	no	yes	Unknown	no	Unknown
Was the dropout rate described and were the characteristics of dropouts compared with the completers?		yes	yes	yes	yes	yes	yes	No (no comparison)	no
Was long-term follow-up in the groups comparable?		no	no	yes	yes	no	no	yes	yes
Was the timing of the outcome measurements in the groups comparable?		yes	yes	yes	yes	yes	no	yes	yes
Was the sample size of each group described by means of a power calculation?		no	yes	yes	yes	yes	no	no	yes
Did the analysis include intention-to-treat analysis?		yes	yes	no	yes	yes	Unknown	no	yes
Were point estimates and measures of variability presented for the primary outcome measures?		yes	Mean but no standard deviation	yes	yes	yes	Mean but no standard deviation	yes	yes

### Methods

Tables 2 and 3 provide an overview of the methods used in the studies, detailing the intervention, control group and participant characteristics as well as the diagnosis addressed by each study. Furthermore, the time points of assessments are listed and rates of abstinence and dropout are reported. The eight included studies all were published in English and described different interventions using the internet as a mode for delivery of treatment or self-help support. Three of the studies focused on bulimia nervosa [14,15,18], two on full or sub-threshold criteria for binge eating disorder [12,13], while the remaining three studies addressed more than one kind of eating disorder [16,17,19]. Eating disorders were diagnosed according to DSM-IV criteria in all but two studies [15,18]. While six of the studies were randomised controlled trials [12,15-19], two studies were controlled but not randomised [13,14]. Of the studies included in this review, two did not assess the stability of effects [14,17] while in the remaining studies the time frame for follow-up ranged between 8 weeks [15] and 12 months after end of the treatment [18].

**Table 2:** Characteristics of treatment programs applied in included studies

**Authors**	**Ljotsson et al. **[16]	**Robinson & Serfaty **[17]	**Johnston et al. **[15]	**Carrard et al. **[12]	**Sanchez-Ortiz et al. **[19]	**Fernandez-Aranda et al. **[14]	**Carrard et al. **[13]	**Ruwaard et al. **[18]
**Type of study/** **Randomisation**	RCT: based on generated random numbers; Stratification procedure was implemented with regard to diagnosis and severity	RCT: randomization was based on generated random numbers	RCT: No exact description was given	RCT: Randomization was based on generated random numbers	RCT: based on generated random numbers; Stratification procedure was implemented with regard to diagnosis and recruitment site	CT: participants were consecutively assigned to either the treatment group or the control group	CT: participants were either offered to take part in the treatment program or asked to participate in the control group during information session for WLT	RCT: randomization was based on generated random numbers
**Intervention**	Self- help based on CBT using the Swedish translation of the book “Overcoming Binge Eating”	Email therapy based on CBT working on a model of the eating disorder	20 minutes writings on the basis of the Pennebaker task	Guided self-help treatment program consisting of 11 modules based on CBT targeting behavioural and psychological aspects of BED	guided self-help treatment program “Overcoming Bulimia Online” consisting of eight sessions based on CBT	Guided self-help program introducing CBT and psychoeducational concepts in seven sequential stepsc	Guided self-help program and consisting of 11 modules based on CBT targeting behavioural and psychological aspects of BED	Guided self-help program referring to CBT main principles for treatment of BN
**Anonymity**	No: EDE interview was performed as main assessment	Yes: no interview or face-to-face meeting was conducted	Yes: no interview or face-to-face meeting was conducted	three additional face-to-face evaluations during a year	introduction in a face-to-face meeting or as telephone assessment	No: two face-to-face evaluations with their coaches during therapy	no: three additional face-to-face evaluations	Yes: only an interview with a diagnostician
**Lengths**	12 weeks	Three months	Three days	Six months	8 to 12 weeks	Four months	Six months	20 weeks
**Frequency of contact**	One to two email contacts per week	On average two emails per week were expected	No contact at all during therapy	Weekly e-mail contact during the intervention phase; monthly e-mail contact during the follow-up period	Therapists sent emails once every one to two weeks and responded to any email received	Weekly e-mail contact with their coach during intervention phase	Therapists sent weekly e-mails and participants had to write at least one email each week to their coaches	Treatment includes 25 scheduled therapist feedback moments
**Number and diagnosis of participants**	73 participants with full or sub-threshold Bulimia Nervosa (BN) or Binge Eating Disorder (BED) diagnosis according to DSM-IV; sub-threshold BN was defined as episodes of binge eating and compensatory behaviour at least twice-monthly for the last three months. BED participants needed to have at least two OBEs per month for the last six months	97 participants suffering from BN (n = 36 purging; n =15 non-purging), BED (n = 26) or EDNOS (n = 20) diagnosis according to DSM-IV	94 participants suffering from BN; Participants were required to score at or above the medium-range cut off for bulimic symptomatology	74 participants suffering from BED (n = 43) or sub-threshold BED (n = 31) diagnosis according to DSM-IV; for sub-threshold BED participants needed to have at least one OBE weekly for the last three months	76 students suffering from BN (n = 39) or EDNOS (n = 37) diagnosis according to DSM-IV; persons suffering from BED were excluded	62 participants suffering from BN purging subtype; diagnosis according to DSM-IV	42 obese participants suffering from BED (n = 21) or sub-threshold BED (n = 21); diagnosis according to DSM-IV; Frequency of binges had to be at least for once a month during the last three months	105 participants suffering from BN symptoms (80% engaged in purging behaviour)
								A formal diagnosis of BN was not an inclusion criteria. Participants had to report recurrent binge eating, inappropriate weight-control behaviour and elevated concern with body shape and weight
**Women N (%)**	69 (97.3%)	93 (95.9%)	71 (75.5%)	74 (100%)	75 (98.7%)	62 (100%)	42 (100%)	104 (99%)
**Age of participants M (SD)**	Intervention Group: 35.5 (11.4)	Whole sample: 24.5 (23–25.9)	Whole sample: 28.9 (9.8)	Whole sample: 36 (11.4)	Whole sample: 23.9 (5.69)	Whole sample: 23.7 (3.6)	Intervention Group: 44.6 (11.4)	Online-CBT: 30 (10)
	Control group: 33.7 (9.3)						Control group: 41.0 (8.2)	Bibliotherapy: 31 (9)
								Waiting list group: 32 (11)

*RCT* Randomized Controlled Trial, *CT* Controlled Trial, *CBT* Cognitive Behavioural Therapy, *EDE* Eating Disorder Examination, *BN* Bulimia Nervosa, *BED* Binge Eating Disorder, *EDNOS* Eating Disorder Not Other Specified.

**Table 3:** Control group characteristics, Outcome measures, time points of assessment, dropout and abstinence rates of included studies

**Authors**	**Ljotsson et al. **[16]	**Robinson & Serfaty **[17]	**Johnston et al. **[15]	**Carrard et al. **[12]	**Sanchez-Ortiz et al. **[19]	**Fernandez-Aranda et al. **[14]	**Carrard et al. **[13]	**Ruwaard et al. **[18]
**Control group**	Waiting list (n = 36) with assessment at mid-treatment and short weekly reports on eating behaviour	Unsupported self directed writing (n = 34). Waiting list control (n = 27)	Superficial writing control group (n = 46)	Waiting list control group (n = 37) with monthly email contact during waiting period	Waiting list control group (n = 38)	Waiting list (n = 31) for 12 weeks keeping a food diary, recording binge eating and purging episodes	Waiting list control group (n = 20), waiting for a weight loss treatment	Waiting list control group (n = 35). Bibliotherapy (n = 35)
**Measures**	EDE-Q	BITE	BITE	EDE-Q	EDE	EDI	EDE-Q	EDE-Q
	EDI-2			EDI-2		BITE		
						Food Diary		
**Assessments**	Pre-treatment	Baseline	Baseline	Baseline	Baseline	Baseline	Baseline	Baseline
	Post-treatment	Post-treatment	Four weeks follow-up	Post-treatment	Post-treatment	Post-treatment	Post-tretament	Post-tretament
	Six month follow-up		Eight weeks follow-up	Six month follow-up	Three month follow-up		Six month follow-up	12 month follow-up
**Treatment dropout**	31.4%	47.2%	16.7%	24.3%	21.1%	45.0%	9%	25.7%
**Study dropout**	2.9%	37.1%	14.9%	17.6%	11.8%	n.a.	4.8%	21.0%
**Definition of abstinence**	no episodes of binge eating or purging during the 28 days prior to the post-treatment assessment	Criteria for an eating disorder are no longer fulfilled	Participants not falling within the clinical range of the BITE total score	Abstinence from OBEs as measured by the EDE-Q for the last 28 days	Lack of vomiting and laxative abuse abstinence from OBEs over a month long period	Abstinence of binges and vomits: no information about duration available	No binge episode over the last three months	No binge or purging episode over the last 28 days according to EDE-Q
**Rate of abstinence** **(Intervention group)**	37%	23.5%	23.7% (at baseline) to 35% (at 8 week follow-up)	35.1%	25.8%	22.6%	45%	Bingeing: 57% (at baseline) to 94% at post-treatment
								Purging: 60% (at baseline) to 100% at post-treatment
**Rate of abstinence (Control group)**	15%	0.0%	n.a.	8.1%	13.9%	0%	15%	Bingeing: 77% (at baseline) to 86% at post-treatment
								Purging: 83% (at baseline) to 89% at post-treatment

*EDE-Q* Eating Disorder Examination Questionnaire, *EDE* Eating Disorder Examination, *EDI-2* Eating Disorder Inventory - 2, *BITE* Bulimia Investigatory Test Edinburgh.

*OBE* Objectivte Binge Episodes as assessed with the Eating Disorder Examination Questionnaire or the Eating Disorder Examination Interview.

### Participants’ characteristics

The included studies involved a total of N = 609 participants suffering from serious eating issues or diagnosed eating disorders, with sample sizes ranging from 42 [13] to 97 [17]. About 97% of the total sample was female. Five of the studies included both genders [15-19] while three only addressed women [12-14]. The average age of participants ranged between 23.7 and 44.6 years.

### Intervention characteristics

Cognitive behavioural therapy (CBT) or modules of CBT formed the basis of all but one intervention [15]. This exception involved an expressive writing task that, according to Pennebaker [50], incorporated exploration of the patients’ thoughts and emotions. Most of the approaches additionally implemented psychoeducational elements. Treatment programs differed in length, ranging from three days for the shortest [15] up to six months for the two longest [12,13]. In brief, six studies offered the patients a guided self-help intervention [12-14,16,18,19], one utilised a specific writing task [15] and one used e-mail therapy that did not follow a structured treatment program [17]. Five of the six studies that used guided self-help interventions developed and used structured treatment programs [12-14,18,19], while in one study [16] the self-help intervention utilised a book with accompanying tasks and homework. The extent of therapist support or guidance in the various internet-based interventions differed from no support at all [15] to an average of two emails per week over the course of the treatment period [17]. In most studies, an average of one contact per week between coaches and participants was planned.

### Control group characteristics

Four studies utilised a comparison group that was a waiting list control group with no professional contact at all [13,17-19]. In three studies, waiting list control groups received exercises to do while waiting [12,14,16] and two of the studies included an additional control group [17,18]. Finally, the study using the expressive writing task as intervention program [15] did not use a waiting list control group at all, but advised participants in the control group to write about superficial topics, in a factual manner, without exploring thoughts or feelings.

### Outcomes

#### Outcomes related to eating disorders

Table 4 illustrates the effect sizes for primary and secondary outcomes, as well as the corresponding CIs. In two studies [12,18], means and standard deviations for the calculation of effect sizes were available in the form of ITT data, while four studies [13,15,16,19] reported these in terms of completers’ data. The remaining two studies did not provide the information required for calculating effect sizes. For one study [14], no standard deviations or test-values were given. Here effect sizes for between groups were taken from reported study results and reported confidence intervals for mean differences were considered. For the other study that did not provide standard deviations [17], F-values were reported but estimation of effect sizes were not possible as the study design included three groups. Here, results of analyses of variance reported in the study were considered instead.

**Table 4:** Effect sizes and confidence intervals of primary and secondary outcomes

		**Ljotsson et al. **[16]	**Johnston et al. **[15]	**Carrard et al. **[12]	**Sanchez-Ortiz et al. **[19]	**Fernandez-Aranda et al. **[14]	**Carrard et al. **[13]	**Ruwaard et al. **[18]
**Primary outcome**								
Binge episodes	ES_within_(CI 95%)	**0.98 (0.58-1.38)**		**1.05 (0.65-1.45)**	**0.75 (0.39-1.11)**		**0.90 (0.40-1.40)**	**1.05 (0.63-1.47)**
	ES_between_(CI 95%)	**0.52 (0.04-1.00)**		**0.52 (0.06-0.98)**	0.35 (-0.10-0.80)	0.49	0.20 (-0.41-0.81)	0.43 (-0.04-0.90)
Purging	ES_within_(CI 95%)	**0.41 (0.06-0.76)**			**0.56 (0.22-0.90)**			**0.77 (0.39-1.15)**
	ES_between_(CI 95%)	**1.63 (1.08-2.18)**			0.27 (-0.18-0.72)			0.45 (-0.02-0.92)
Vomiting	ES_within_(CI 95%)				**0.46 (0.12-0.80)**			
	ES_between_(CI 95%)				0.29 (-0.16-0.74)	**0.78**		
**Secondary Outcome**								
**EDE-Q**								
Restraint	ES_within_(CI 95%)	0.32 (-0.02-0.66)		**0.47 (0.13-0.81)**	**1.26 (0.83-1.69)**		0.23 (-0.19-0.65)	
	ES_between_(CI 95%)	0.26 (-0.21-0.73)		0.14 (-0.32-0.60)	**0.79 (0.32-1.26)**		0.24 (-0.37-0.85)	
Eating concern	ES_within_(CI 95%)	**0.86 (0.47-1.25)**			**1.05 (0.65-1.45)**		**0.78 (0.30-1.26)**	
	ES_between_(CI 95%)	**0.99 (0.49-1.49)**			**0.84 (0.37-1.31)**		0.39 (-0.22-1.00)	
Shape concern	ES_within_(CI 95%)	**0.51 (0.16-0.86)**		**0.89 (0.51-1.27)**	**1.02 (0.63-1.41)**		**1.02 (0.50-1.54)**	
	ES_between_(CI 95%)	**0.92 (0.42-1.42)**		0.28 (-0.18-0.74)	**1.05 (0.57-1.53)**		0.58 (-0.04-1.20)	
Weight concern	ES_within_(CI 95%)	**0.78 (0.40-1.16)**			**0.73 (0.37-1.09)**		**1.11 (0.57-1.65)**	
	ES_between_(CI 95%)	**1.10 (0.59-1.61)**			**0.79 (0.32-1.26)**		0.18 (-0.43-0.79)	
Total	ES_within_(CI 95%)	**0.71 (0.34-1.08)**		**1.18 (0.76-1.60)**	**1.21 (0.79-1.63)**		**0.79 (0.31-1.27)**	**1.18 (0.75-1.61)**
	ES_between_(CI 95%)	**0.96 (0.46-1.46)**		0.45 (-0.01-0.91)	**1.09 (0.61-1.57)**		0.32 (-0.29-0.93)	**0.50 (0.02-0.98)**
**EDI (-2)**								
Drive for thinness	ES_within_(CI 95%)	**0.55 (0.19-0.91)**		**0.62 (0.27-0.97)**				
	ES_between_(CI 95%)	**0.99 (0.49-1.49)**		**0.48 (0.02-0.94)**		0.25		
Body dissatisfaction	ES_within_(CI 95%)	**0.39 (0.05-0.73)**		**0.60 (0.25-0.95)**				
	ES_between_(CI 95%)	**0.76 (0.27-1.25)**		0.05 (-0.41-0.51)		0.16		
Interoceptive awareness	ES_within_(CI 95%)	**0.70 (0.33-1.07)**		**0.53 (0.18-0.88)**				
	ES_between_(CI 95%)	**0.88 (0.39-1.37)**		**0.46 (0.00-0.92)**		0.03		
Bulimia	ES_within_(CI 95%)	**0.79 (0.41-1.17)**		**1.56 (1.08-2.04)**				
	ES_between_(CI 95%)	**0.94 (0.44-1.44)**		**1.08 (0.59-1.57)**		0.54		
Interpersonal distrust	ES_within_(CI 95%)	0.02 (-0.31-0.35)		0.21 (-0.12-0.54)				
	ES_between_(CI 95%)	0.00 (-0.47-0.47)		0.17 (-0.29-0.63)		0.32		
Ineffectiveness	ES_within_(CI 95%)	**0.47 (0.12-0.82)**		**0.36 (0.03-0.69)**				
	ES_between_(CI 95%)	**0.57 (0.09-1.05)**		0.33 (-0.13-0.79)		0.05		
Maturity fears	ES_within_(CI 95%)			0.23 (-0.10-0.56)				
	ES_between_(CI 95%)			0.15 (-0.31-0.61)		**0.63**		
Perfectionism	ES_within_(CI 95%)	0.21 (-0.13-0.55)		0.12 (-0.20-0.44)				
	ES_between_(CI 95%)	0.22 (-0.25-0.69)		0.19 (-0.27-0.65)		0.13		
Impulse regulation	ES_within_(CI 95%)			**0.51 (0.17-0.85)**				
	ES_between_(CI 95%)			0.31 (-0.15-0.77)				
Social insecurity	ES_within_(CI 95%)			**0.64 (0.29-0.99)**				
	ES_between_(CI 95%)			0.27 (-0.19-0.73)				
Total	ES_between_(CI 95%)					0.37		
**BITE**								
Severity	ES_within_(CI 95%)		0.14 (-0.14-0.42)					
	ES_between_(CI 95%)		0.28 (-0.13-0.69)					
Symptom	ES_within_(CI 95%)		0.10 (-0.18-0.38)					
	ES_between_(CI 95%)		0.03 (-0.37-0.43)					
Total	ES_within_(CI 95%)		0.14 (-0.14-0.42)					
	ES_between_(CI 95%)		0.13 (-0.28-0.54)			**1.03**		

*ES* Effect Sizes calculated according to Hedges [40], *CI* Confidence intervals were calculated using formulas according to Hedges and Olkin [40]. Boldface data show CIs not covering zero.

*EDE-Q* Eating Disorder Questionnaire, *EDI-2* Eating Disorder Inventory - 2, *BITE* Bulimia Investigatory Test Edinburgh.

#### Primary outcomes

**Figure 2:**
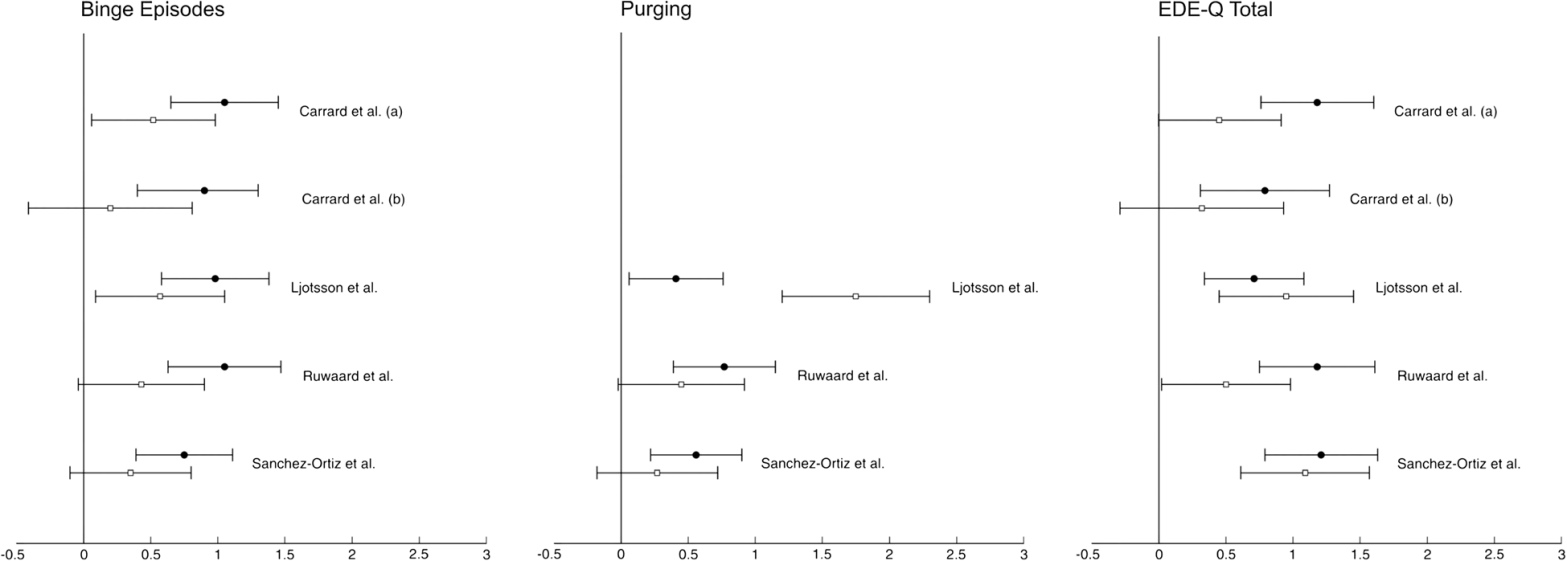
Effect sizes and corresponding confidence intervals for Bingeing, Purging and the EDE-Q Total Score.

Figure 2 shows effect sizes and corresponding confidence intervals for bingeing and purging. Six of the studies assessed bingeing or purging, while in two studies [15,17] these behaviours were not assessed. Medium to large effect sizes from pre- to post-treatment were found in the intervention group for both bingeing and purging episodes, ranging from 0.75 to 1.05 for binge episodes and from 0.41 to 0.77 for purging. When effect sizes were calculated between groups, however, a significant reduction in the number of binge episodes in the intervention group as compared to the control group was only found in two studies [12,16], with moderate effect sizes. In studies that assessed purging behaviour, no significant differences in reduction of purging between groups were found, with one exception. One study [16] found a significant reduction in purging behaviour in the intervention group compared to the control group, with a considerably high effect size of 1.63. Sanchez-Ortiz and colleagues [19] additionally assessed frequency of vomiting and found medium-sized effects within the treatment group, while effects were only small when the two groups were compared. In contrast to this, Fernandez-Aranda et al. [14] assessed frequency of vomiting and reported high effect sizes after comparing the two groups.

The definition of abstinence differed between studies and the rates of abstinence in the intervention groups were found to vary widely in the different studies between 22.6% [14] and 45% [13]. While in most studies, abstinence was defined as absence of the relevant eating disorder behaviour (e.g. bingeing or purging) over a defined time period, in two studies abstinence was defined as no longer falling within the clinical range of the BITE [15] or no longer fulfilling criteria for an eating disorder according to the DSM-IV [17]. All of the studies found a higher rate of abstinence in the intervention group compared to the control group with lowest contact, but this difference was only tested for significance in four studies [12,13,17,18]. All of these analyses reached statistical significance.

#### Secondary outcomes

Figure 2 also shows effect sizes and corresponding confidence intervals for EDE-Q total. Regarding secondary outcomes, as assessed with the questionnaires described above, the included studies found high effects from pre- to post-treatment in the EDE-Q total, as assessed in five studies [12,13,16,18,19]. Positive results of the intervention were also found for the subscales of the EDE-Q, where assessed. When groups were compared for EDE-Q total, moderate to high effect sizes were found in three studies [16,18,19], while in the two studies of Carrard and colleagues [12,13] these effect sizes were only small to moderate, as were the results of the EDE-Q subscales. In the remaining two studies assessing EDE-Q subscales [16,19], effect sizes between groups were high with the one exception in the “Restraint” subscale, that was only significant in the study conducted by Sanchez-Ortiz et al. [19]. In two studies [12,16] the EDI-2 was implemented in addition to the EDE-Q to aid treatment outcome assessment. In both studies medium to high effects of the intervention were found from pre- to post-treatment for most of the subscales, reinforcing the results reported for the EDE-Q. These studies also showed medium to high effect sizes on some subscales of the EDI-2 when groups were compared. Additionally, in the study by Fernandez-Aranda [14], the effect sizes reported were small to medium. In two of the studies – those conducted by Robinson and Serfaty [17] and Johnston et al. [15] – eating disorder symptoms were not assessed with either the EDE-Q or the EDI but with the Bulimia Investigatory Test Edinburgh (BITE) [39]. No significant treatment effects either within or between the treatment groups could be shown in the study of Johnston et al. [15]. In the study of Robinson and Serfaty [17], only changes within the whole group of participants were examined. For this sample, no significant changes in questionnaire scores were found.

#### Dropout rates

As authors defined dropout differently between studies, treatment dropouts were considered separately from study dropouts and rates are displayed in Table 3. Treatment dropouts were intervention group participants who did not complete the treatment, whereas study dropouts were participants from either group who did not fill out the post-treatment assessment. While treatment dropout rates were between 9% [13] and 47.2% [17], study dropouts ranged between 2.9% [16] and 37.1% [17]. In this study, no information was given about whether the number of participants, who did not complete treatment, differed from the number of participants, who did not fill out post-assessment, meaning that the treatment dropout we report here is identical to study dropout in the treatment group. In two studies [12,19] treatment dropouts referred to participants who did not use treatment at all or did not finish the first module of treatment.

#### Stability of results

Of the six studies that included follow-up measures [12,13,15,16,18,19], the one with the 8-week follow-up failed to find stable treatment effects [15]. The remaining five studies found that the results achieved at post-treatment were stable or even improved over the follow-up period.

### Additional outcomes

Table 5 gives an overview of the additional results. In two studies [14,18], neither depression nor any other additional outcome was explicitly assessed. All of the remaining studies assessed depression: three [12,13,17] utilising the Beck Depression Inventory (BDI) [42], or BDI-II [43], two studies [15,19] making use of the Hospital Anxiety and Depression Scale (HADS) [44,45] and one study [16] using the Montgomery Åsberg Depression Scale Self-assessment (MADRS) [46]. Divergent results were found between the studies. Again, in the study by Robinson and Serfaty [17], no information for calculating effect sizes was available and no significant differences were found for the analysis of variance performed. In terms of effect sizes within the intervention group, four studies found medium to high effect sizes for depression [12,13,16,19], while no effects were found for the three day writing task study by Johnston et al. [15]. For three of the studies [13,16,19], significant differences were also found between the groups. As they applied the HADS, two studies [15,19] were additionally able to assess anxiety as an outcome variable. The study conducted by Johnston et al. [15] did not find effects within or between groups, but in the study by Sanchez-Ortiz et al. [19], reduction of anxiety resulted in high effect sizes both within and between the groups. Quality of life or satisfaction with life were assessed in four studies [12,13,16,19]. Here, medium- to high-sized effects were seen for the intervention group, with exception of one study [12]. These results did not translate to a medium to high between-group effect for the study by Ljotsson et al. [16], however.

**Table 5:** Effect sizes and confidence intervals of questionnaires regarding additional outcomes

		**Ljotsson et al. **[16]	**Johnston et al. **[15]	**Carrard et al. **[12]	**Sanchez-Ortiz et al. **[19]	**Carrard et al. **[13]
**Depression**						
BDI (-II)	ES_within_ (CI 95%)			**0.61 (0.26-0.96)**		**0.61 (0.15-1.07)**
	ES_between_ (CI 95%)			0.37 (-0.09-0.83)		**0.80 (0.17-1.43)**
HADS	ES_within_ (CI 95%)		0.08 (-0.20-0.36)		**2.18 (1.59-2.77)**	
	ES_between_ (CI 95%)		0.18 (-0.23-0.59)		**0.97 (0.49-1.45)**	
MADRS	ES_within_ (CI 95%)	**0.78 (0.40-1.16)**				
	ES_between_ (CI 95%)	**0.76 (0.27-1.14)**				
**Anxiety**						
HADS	ES_within_ (CI 95%)		0.27 (-0.02-0.56)		**1.01 (0.62-1.40)**	
	ES_between_ (CI 95%)		0.02 (-0.38-0.42)		**0.82 (0.33-1.31)**	
**Quality of life**						
*IWQOL*-*Lite*						
Physical functioning	ES_within_ (CI 95%)					**0.44 (0.00-0.88)**
	ES_between_ (CI 95%)					0.37 (-0.24-0.98)
Self-esteem	ES_within_ (CI 95%)					**0.55 (0.10-1.00)**
	ES_between_ (CI 95%)					0.59 (-0.03-1.21)
Sexual life	ES_within_ (CI 95%)					**0.49 (0.05-0.93)**
	ES_between_ (CI 95%)					**0.84 (0.21-1.47)**
Public distress	ES_within_ (CI 95%)					**0.52 (0.07-0.97)**
	ES_between_ (CI 95%)					**0.73 (0.10-1.36)**
Work	ES_within_ (CI 95%)					0.43 (-0.01-0.87)
	ES_between_ (CI 95%)					**0.94 (0.30-1.58)**
Total score	ES_within_ (CI 95%)			0.30 (-0.03-0.63)		**0.68 (0.21-1.15)**
	ES_between_ (CI 95%)			0.01 (-0.45-0.47)		**0.78 (0.15-1.41)**
*WHOQOL*-*Bref*						
Physical health	ES_within_ (CI 95%)				**1.01 (0.62-1.40)**	
	ES_between_ (CI 95%)				**1.03 (0.55-1. 51)**	
Psychological health	ES_within_ (CI 95%)				**0.77 (0.41-1.13)**	
	ES_between_ (CI 95%)				**0.80 (0.33-1.27)**	
Social	ES_within_ (CI 95%)				**0.67 (0.32-1.02)**	
	ES_between_ (CI 95%)				**0.65 (0.19-1.11)**	
Enviromental	ES_within_ (CI 95%)				0.15 (-0.17-0.47)	
	ES_between_(CI 95%)				0.19 (-0.26-0.64)	
SWLS						
Total	ES_within_ (CI 95%)	**0.41 (0.06-0.76)**				
	ES_between_ (CI 95%)	0.31 (-0.17-0.79)				

*ES* Effect Sizes calculated according to Hedges [40], *CI* Confidence intervals were calculated using formulas according to Hedges and Olkin [40]. Boldface data show CIs not covering zero.

*BDI* (-II) Beck Depression Inventory, *HADS* Hospital Anxiety and Depression Scale, *MADRS* Montgomery Åsberg Depression Scale Self-assessment, *SWLS* Satisfaction with Life Scale; IWQOL-Lite, Impact of Weight on Quality of Life, *WHOQOL-Bref* WHO Quality of Life Questionnaire (short form).

## Discussion

This review systematically evaluated the efficacy of internet-based treatment programs for different eating disorders in participants aged at least 16, based upon evidence from controlled studies. An article focusing on the effectiveness of cognitive-behavioural guided self help for eating disorders has recently been published [51], but to our knowledge this study is the first review examining the standardized effects of interventions delivered through the internet on participants suffering from different kinds of eating disorders. All studies included in this review were published in the last six years, since 2007, highlighting that this research field is relatively novel. For five of the eight studies included in this review, good methodological quality was noted [12,16-19].

All but one intervention [15] were based on CBT, which emphasizes the suitability of cognitive behavioural methods as a basis for the development of internet-based treatments for eating disorders. Of the seven studies based on CBT, six offered patients a guided self-help intervention [12-14,16,18,19]. Of these, one delivered the self-help intervention by book, with accompanying tasks and homework [16], while the other studies developed and used a structured treatment program. The remaining CBT-based study used e-mail therapy without following a structured treatment program [17]. All studies that were based on CBT principles and provided relevant information to calculate effect sizes found significant reductions of eating disordered behaviour within the intervention groups from pre- to post-treatment for primary outcomes (e.g. bingeing and purging) as well as secondary outcomes. These interventions were also found to be beneficial in comparison to being placed on a waiting list. The findings applied to the self-help programs implemented over the internet, but also for the self help book intervention with e-mail support and for e-mail-therapy. In summary, the results suggest that a variety of treatments, based on CBT and using the internet, can help to reduce symptoms related to eating disorders such as bulimia nervosa, binge eating and EDNOS.

There was high variance in the rates of abstinence between studies. Interestingly, the three studies with the highest rates of abstinence had the most conservative time criteria. Since these studies included patients suffering from binge eating disorder [12,13,16] results are in line with previous meta-analysis findings from two other studies. In one study, the odds ratios for abstinence rates in a RCT addressing binge eating disorder significantly improved after psychotherapy and structured self-help [52]. A second study compared guided and unguided self-help for binge eating [53], where relatively high remission rates were found for OBEs. These findings can be interpreted with respect to the high spontaneous remission found for binge eating [54]. In this review, benefits of the guided self-help interventions based on CBT were superior to those elicited by the e-mail therapy [17] or the three day writing task [15].

Additionally, effects on secondary outcomes, as assessed by questionnaires, were comparable with those produced by face-to-face therapies. For example, a meta-analysis examining the effects of different treatments for binge eating [52] found medium to high effect sizes for psychotherapy as well as structured self-help, both mainly based on CBT. These results are in line with significant medium to high effect sizes found in the studies included in this review that addressed patients suffering from BED [12,13,16]. Furthermore, a meta-analysis of studies that used CBT in face-to-face treatment for bulimia nervosa found effect sizes ranging from −0.03 to 1.00 for behavioural measures and from 0.26 to 0.98 for cognitive measures [55]. Again, these results are in line with results found in the CBT-based intervention for BN of studies included in the review. Additionally, follow-up examination results indicated that the effects of the treatments did not decrease over time, implying long-term stability of these positive effects of CBT-based guided self-help interventions for eating disorders delivered over the internet. The two studies that did not find significant effects or stable treatment results either within or between groups were those by Johnston et al. [15] and Robinson and Serfaty [17]. Neither study utilized structured self-help based upon CBT principles, instead using e-mail therapy [17] or a writing task [15]. Furthermore, the study by Johnston et al. [15] differed from the other studies in terms of lengths and therapeutic contact, and one could argue that the focus was not the internet as a tool, but rather evaluation of an intervention paradigm which had not previously been assessed for the treatment of eating disorders.

To further evaluate internet-based interventions for the treatment of eating disorders, dropout rates should be taken into account. The relative numbers of participants who did not finish treatment (treatment dropout) differed between the studies included and were higher in studies that included participants suffering from symptoms of BN [14,16,18,19]. This matches results from face-to-face therapies, where high treatment dropout rates have also been documented. While Garner [56] reported a mean dropout rate of 15.3% in their review of CBT for the treatment of bulimia nervosa, the dropout rates reported in controlled studies are assumed to be an underestimate of the rates in a general clinical setting [57-61]. One possible explanation for the different dropout rates that were found in internet-based interventions of depression and anxiety [62] is the level of anonymity. But, contrary to this finding, no clear connection between anonymity and treatment dropout was seen in the present review. The studies providing anonymity were not necessarily those where high treatment dropout rates were reported. Instead of anonymity, diagnosis seems to be more relevant to dropout rates. So, dropout rates found in the studies addressing exclusively participants who showed (sub-threshold) binge eating behaviour [12,13] were relatively low, at 24.3% and 9%. These dropout rates are in accordance with studies of non-internet-based self-help techniques for those patients [63]. It is hypothesized that patients suffering from binge eating are highly motivated to work on their eating problems due to the association of binge eating with high psychological impairment and related health problems [64,65]. Apart from anonymity and diagnosis, it is worth noting that dropout rates for internet-based interventions in other psychiatric disorders generally differ widely. For instance, Titov and colleagues [66] found a dropout rate of 11% in a trial of clinician-assisted internet-delivered CBT for depression, while Spek et al. [31] reported a dropout rate of 66% for internet-delivered CBT for sub-threshold depression.

In the literature, several different factors (e.g. duration of treatment and amount of contact) have been found to be associated with differences in treatment effects. For example, the influence of therapist support on treatment outcomes for depression was found to be strong [26,27]. Furthermore, guided self-help has been shown to produce larger treatment effects than pure self-help in BED [53,67] and providing guidance might increase both adherence to and the benefits of computerized interventions [68]. Unfortunately, it was not possible to draw conclusions in this review about how much influence the duration of an internet-based intervention or the amount of contact with the coaches has on its efficacy, since variability of these two factors was limited between studies. When combined with the different kinds of treatments and participants addressed, no clear conclusion can be drawn.

Although the internet-based interventions discussed in this review were not specifically aimed at reducing comorbid symptoms such as depression, anxiety or at increasing quality of life, these can be assumed to be relevant factors for eating disorder patients. Overall results of the present review indicate that depressive symptoms and anxiety can be reduced by internet-based interventions. This is in line with previous data from face-to-face studies [52]. For example in their meta-analysis, Vocks and colleagues found significant but small mean effect sizes for depression after psychotherapy and structured self-help when comparing experimental and control groups. It can be assumed that improvement of eating disorder symptoms might influence depressiveness and symptoms of anxiety. Furthermore, significant treatment effects found in this review indicate that quality of life and satisfaction with life are related to a reduction of eating disorder symptoms.

### Limitations

Several limitations of this review have to be addressed. For example, only a limited number of studies could be included. The fact that these studies used different kinds of interventions and addressed different eating disorders complicated the process of reaching reliable conclusions. To include more articles, eligibility criteria would have had to be broader, but increasing the variability of the analysed treatment programs, for instance by including prevention programs, would have lead to even less comparability of results. One recommendation would be to carry out the literature search in more databases to assure the inclusion of all relevant articles. But as internet-based interventions for eating disorders are a relatively novel treatment option still undergoing research, we suspect that the electronic search and the subsequent screening of the reference lists described above should lead to the inclusion of all articles relevant for the review.

Finally, the absence of a CBT-based program, the short duration of the intervention and the lack of post-treatment assessment made the study conducted by Johnston et al. [15] difficult to compare with the other studies in the review. Nonetheless, this study fulfilled all selection criteria and therefore needed to be included in this review. Furthermore, although effect sizes of internet-based interventions can be considered medium to high, comparing them to face-to-face interventions will always be difficult, especially with regard to various sample characteristics (e.g. inpatient vs. outpatient). Generally, further research should evaluate the characteristics of samples recruited for internet-based interventions for eating disorders.

## Conclusions

In summary, internet-based interventions based upon CBT principles can be assumed to be a good alternative to face-to-face therapies for the treatment of eating disorders. Especially internet-based guided self-help programs and self-help books supported by e-mail contact showed promising results. This conclusion is supported by the fact that effect sizes of these interventions are comparable to face-to-face treatments for eating disorders and the apparent stability of the treatment outcomes. Furthermore, treatments delivered via the internet are especially relevant for patients who do not have access to conventional therapy programs due to different reasons.

Unfortunately, due to the small number of studies, the differences in disorders addressed and assessment methods used in each study, these conclusions must be interpreted as promising but not definitive. Further research investigating different treatments and self-help programs is needed to analyze the different components of CBT and to identify the most effective strategies. Additionally, predictors of treatment outcome should be identified and examined in order to better deduce which treatment program fits best for each patient.

In conclusion, despite promising effects which internet-based interventions utilizing structured self-help based upon CBT principles seem to have on patients suffering from different kinds of eating disorders, further research is needed to identify factors that lead to these positive results.

## Abbreviations

RCT: Randomized controlled trial; CT: Controlled trial; CBT: Cognitive behavioural therapy; BN: Bulimia nervosa; BED: Binge eating disorder; EDNOS: Eating disorder not other specified; EDI: Eating Disorder Inventory; EDE-Q: Eating Disorder Examination Questionnaire; BITE: Bulimia Investigatory Test Edinburgh; BDI: Beck Depression Inventory; HADS: Hospital Anxiety and Depression Scale; MADRS: MontgomeryÅsberg Depression Scale Self-assessment (MADRS); SWLS: Satisfaction with Life Scale; IWQOL-Lite: Impact of Weight on Quality of Life; WHOQOL-Bref: WHO Quality of Life Questionnaire (short form).

## Competing interests

The authors declare that they have no competing interests.

## Authors’ contributions

Manuscript concept and design: RD, AK, BW. Acquisition of data: RD, AT. Analysis and interpretation of data: RD, BW. Drafting of manuscript: RD, AT, AK, BW. Revision of manuscript: RD, AT, AK, BW. All authors read and approved.

## Pre-publication history

The pre-publication history for this paper can be accessed here:

http://www.biomedcentral.com/1471-244X/13/207/prepub
